# Reactivities of Quinone Methides versus *o*-Quinones in Catecholamine Metabolism and Eumelanin Biosynthesis

**DOI:** 10.3390/ijms17091576

**Published:** 2016-09-20

**Authors:** Manickam Sugumaran

**Affiliations:** Department of Biology, University of Massachusetts Boston, Boston, MA 02125, USA; manickam.sugumaran@umb.edu; Tel.: +1-617-287-6598

**Keywords:** catecholamine metabolism, quinone methides, quinone isomerization, eumelanin biosynthesis, dihydroxyindole polymers, quinone reactivity

## Abstract

Melanin is an important biopolymeric pigment produced in a vast majority of organisms. Tyrosine and its hydroxylated product, dopa, form the starting material for melanin biosynthesis. Earlier studies by Raper and Mason resulted in the identification of dopachrome and dihydroxyindoles as important intermediates and paved way for the establishment of well-known Raper–Mason pathway for the biogenesis of brown to black eumelanins. Tyrosinase catalyzes the oxidation of tyrosine as well as dopa to dopaquinone. Dopaquinone thus formed, undergoes intramolecular cyclization to form leucochrome, which is further oxidized to dopachrome. Dopachrome is either converted into 5,6-dihydroxyindole by decarboxylative aromatization or isomerized into 5,6-dihydroxyindole-2-carboxylic acid. Oxidative polymerization of these two dihydroxyindoles eventually produces eumelanin pigments via melanochrome. While the role of quinones in the biosynthetic pathway is very well acknowledged, that of isomeric quinone methides, however, remained marginalized. This review article summarizes the key role of quinone methides during the oxidative transformation of a vast array of catecholamine derivatives and brings out the importance of these transient reactive species during the melanogenic process. In addition, possible reactions of quinone methides at various stages of melanogenesis are discussed.

## 1. Introduction

Tyrosine and its hydroxylated product dopa constitute major precursors for cellular catecholamines in practically all organisms. They serve as the starting point for the biosynthesis of crucial hormones, neurotransmitters, alkaloids, coenzymes, and a plethora of pigments. Melanin is an important phenolic pigment formed from the amino acid tyrosine and its derivatives in nature. It is widely distributed in practically all organisms and is extensively used by animals for pigmentation, camouflage, mimicry, UV protection and thermoregulation [1,2,3,4,5]. There are two kinds of melanins biosynthesized in animals. The yellow to red pheomelanin arises by the oxidative polymerization of cysteinyldopa derivatives formed by the condensation of the amino acid, cysteine and dopaquinone (or its derivatives). The brown to black eumelanin is generated by the oxidative polymerization of 5,6-dihydroxyindoles, which in turn arise by intramolecular cyclization and further transformation of dopaquinone [1,2,3,4,5]. The focus of this review is on eumelanin.

**Figure 1:**
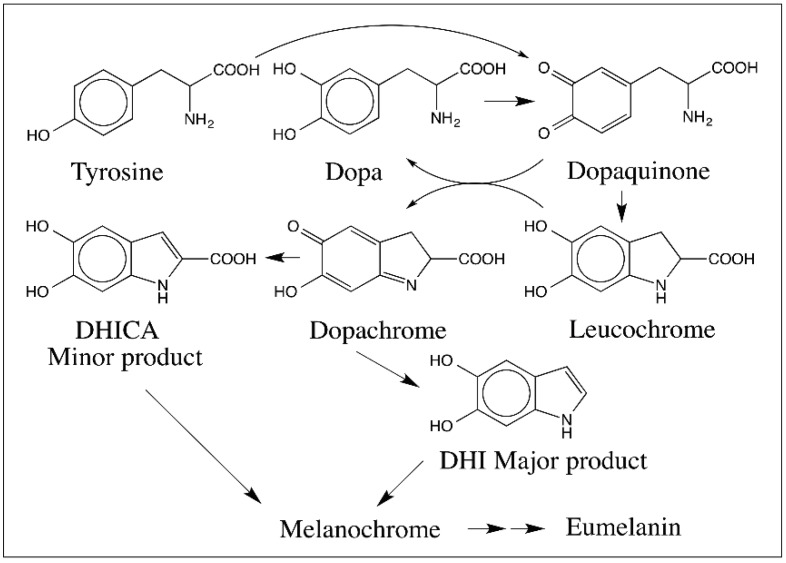
Raper–Mason Pathway for the biosynthesis of eumelanin. Tyrosinase converts tyrosine to dopaquinone via dopa However, dopa is not formed directly from tyrosine but indirectly by the reduction of dopaquinone. Dopaquinone undergoes intrmolecular cyclization producing leucochrome nonenzymatically, which is further oxidized to dopachrome by dopaquinone. Dopachrome is converted to 5,6-dihydroxyindole (DHI) as the major product and 5,6-dihydroxyindole-2-carboxylic acid (DHICA) as the minor product. Further oxidation of DHI to melanochrome and its eventual polymerization leads to melanin pigment.

Raper–Mason pathway for the biosynthesis of eumelanin was established in early 1930s–1940s by the pioneering work of Raper and Mason [6,7,8,9,10]. They studied the enzymatic oxidation of tyrosine and dopa systematically and delineated a pathway for the biosynthesis of eumelanin, which is depicted in Figure 1. According to this pathway, tyrosinase converts tyrosine to dopaquinone via dopa. However, dopa is not directly formed but indirectly produced by the reduction of dopaquinone to dopa. Dopaquinone exhibits a rapid nonenzymatic intramolecular cyclization producing leucochrome, which reduces dopaquinone to dopa and gets oxidized to dopachrome (Figure 1). After the formation of dopaquinone, most of the reaction leading to melanin biosynthesis can occur with out the need for any enzyme. Thus, the common belief was that tyrosinase is the sole enzyme responsible for the production of eumelanin pigment in nature.

Dopachrome was initially believed to undergo transformation to produce 5,6-dihydroxyindole (DHI), as the major product with small amounts of DHICA. The dihydroxyindoles are further oxidized to melanochrome and eventually produces dark colored melanin polymer. However, this simple scheme was modified with the discovery of two new enzymes associated with melanogenic pathway. An enzyme converting dopachrome to 5,6-dihydroxyindole-2-carboxylic acid (DHICA) was first discovered in mammalian systems [11,12,13,14,15,16]. Subsequently a sister enzyme catalyzing the conversion of dopachrome to DHI was characterized from insects [17,18,19]. Although the mammalian enzyme was initially named as dopachrome isomerase, the current accepted name for this enzyme is dopachrome tautomerase (DCT) [13,14]. The sister enzyme in insects converts dopachrome to DHI and hence should be called dopachrome decarboxylase. However, it also converts dopachrome methyl ester to DHICA methyl ester thus catalyzing a perfect tautomerization reaction [19]. Therefore, this enzyme will be referred to as dopachrome decarboxylase/tautomerase (DCDT). Another enzyme named DHICA oxidase that catalyzed the oxidation of DHICA to its corresponding quinone was discovered in mouse [20,21]. With the help of these enzymes, DHI melanin, DHICA melanin and mixed DHI/DHICA eumelanins are formed in various animal systems. Thus, melanin biosynthesis in animals seems to occur with the help of three (or more) enzymes as opposed to one. As shown in Figure 1, quinone reactivity plays a central role in eumelanin biosynthesis. However, less known and more reactive quinone methides, which are isomers of 4-alkylquinones, also participates in key conversions and contributes to the reactivity of different catecholamines associated with eumelanogenic pathway. This review summarizes the importance of quinone methides during the oxidative transformation of a variety of catecholamines related to eumelanin and serves to illustrate their importance.

## 2. Reactions of Quinones

**Figure 2:**
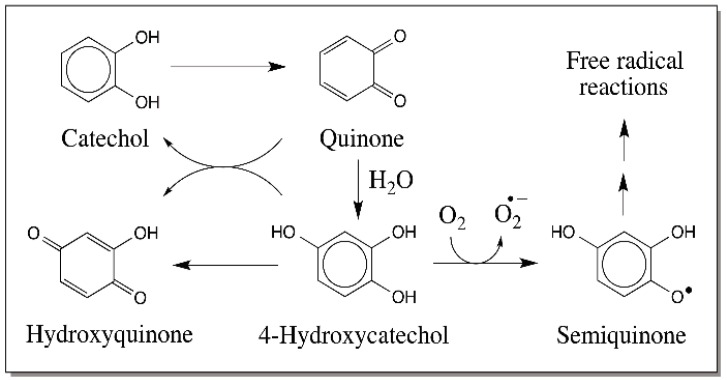
Formation of hydroxylated catechols and their further reactions. *o*-Quinone formed from catechol by two-electron oxidation can undergo addition with water forming hydroxyl catechol, which will readily undergo oxidation to hydroxy-*p*-quinone as well as semiquinone by reaction with molecular oxygen. Eventually, these quinonoid compounds will exhibit polymerization.

Catecholamine derivatives are prone to easy oxidation. Their two-electron oxidation leads to the most common product, 4-alkyl quinones. The reactivities of quinones are extremely vital for the biological role of many catechols. Therefore, the reactions of quinones will be considered first. Only the reactions of *o*-quinones are given in this review and the reactions of *p*-quinones are not covered. The quinonoid nucleus is very reactive and unstable. Hence, it exhibits rapid Michael-1,4-addition reactions with a variety of nucleophiles to generate substituted catechols. The reaction leads to 1,4-adduct of nucleophiles on the ring and ends up with the re-aromatization of the quinone nucleus. Quinones can react with water forming 1,4-addition product. The resultant trihydroxy phenols are not very stable and undergo rapid nonenzymatic aerial oxidation (Figure 2). Reaction with molecular oxygen leads to the production of superoxide anion and semiquinone radicals, which will exhibit radical coupling to produce dimers and other oligomers. Moreover, trihydroxyphenol can also be oxidized to its two-electron oxidation product, hydroxy-*p*-quinone. The importance of the above reaction becomes evident when one looks at the biosynthesis of topaquinone cofactor found in amine oxidases [22,23]. A special tyrosine group in the precursor protein is oxidized to dopa and further converted to 6-hydroxydopa. Aerial oxidation of this trihydroxy compound generates the required topaquinone cofactor in these enzymes [22,23].

**Figure 3:**
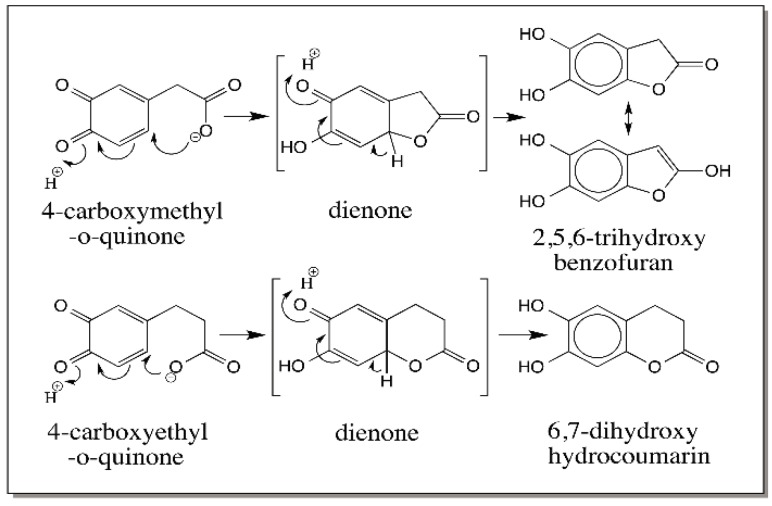
Nucleophilic addition of internal carboxyl group on quinone. Both carboxymethyl-*o*-quinone and carboxyethyl-*o*-quinone exhibit rapid intramolecular cyclization due to the presence of suitably substituted internal carboxyl group yielding cyclic products.

Similar to water reaction, alcohol group also reacts with the quinones forming the Michael-1,4-addition products. The resultant substituted ether does not exhibit any significant reactivity with molecular oxygen but is capable of further oxidation to typical *o*-quinones. Interestingly quinones can also react with the carboxyl groups forming esters. However, this reaction occurs most likely if the carboxyl group is tied up to the quinonoid nucleus in a suitable position such as those found in 4-carboxymethyl-*o*-quinone and 4-carboxyethyl-*o*-quinone [24,25]. Oxidation of both 3,4-dihydroxy phenylacetic acid and 3,4-dihydroxy hydrocinnamic acid leads to the corresponding quinones (Figure 3). The quinones thus formed, exhibit instantaneous intramolecular cyclization generating 2,5,6-trihydroxybenzofuran and 6,7-dihydroxyhydrocoumarin, respectively. The quinones of *N*-acetyl dehydro dopa also exhibit intramolecular cyclization producing coumarin derivatives [26].

**Figure 4:**
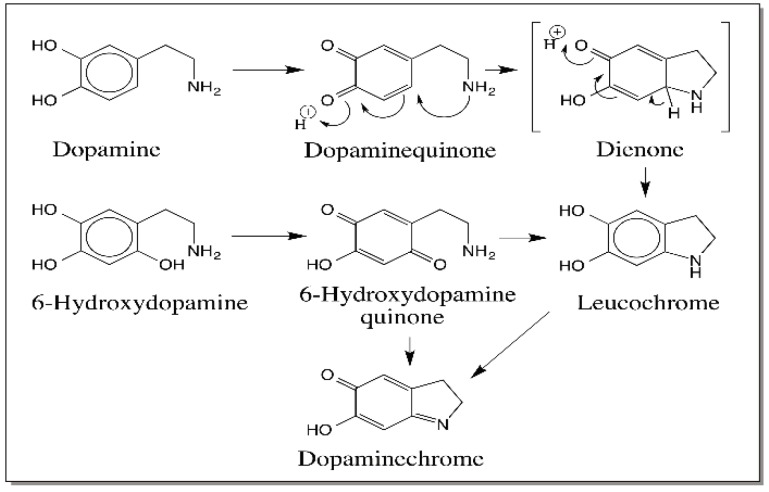
Reactions of quinone with internal amines. Dopamine quinone formed by the two-electron oxidation of dopamine reacts rapidly with the internal amino group forming leucochrome, which is converted readily to dopaminechrome similar to the dopachrome conversion reaction shown in Figure 1. Interestingly dopaminechrome is also generated during the oxidation of 6-hydroxydopamine as a condensation of product of 6-hydroxydopaminequinone.

Importantly, amines—both primary and secondary amines—react under facile conditions with quinones producing amino substituted catechols, which suffer rapid oxidation yielding quinoneimines. As witnessed in the case of carboxyl group, internal amino group will react extremely fast with the quinone forming cyclized product. It is this reaction that in fact forms the central theme in melanin biosynthesis. As shown in Figure 1, dopaquinone undergoes rapid intramolecular cyclization with the internal amino group generating the leucochrome, which is rapidly oxidized by the dopaquinone to dopachrome. Dopamine quinone also exhibits the same reaction (Figure 4) [27]. Furthermore, 6-hydroxydopamine, the hydroxylated product of dopamine quinone, upon oxidation produces the same dopaminechrome as that produced by dopamine Figure 4 [28].

**Figure 5:**
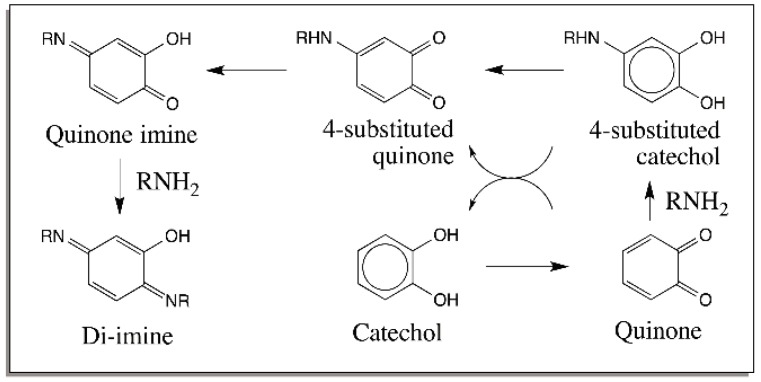
Reactions of quinone with external amines. Quinones formed by the oxidation of catechols react with external amino group forming 4-substituted catechols. These catechols are unstable and form further adducts via substituted quinones.

The above reactions can be translated to external nucleophilic addition of amines also. Figure 5 summarizes the reactions of simple quinone with external amine. Note the remarkable resemblance between the internal and external reactions of amines. In the case of secondary amines, the best characterized reaction being the one that takes place between the imidazole group of histidine and quinone [29]. The addition leads to 4-substituted catechols that do not readily oxidize to quinones. This reaction plays a vital role in insect cuticular sclerotization reaction, which has been shown to remarkably parallel melanin biosynthesis [30,31,32].

Thiols react with quinones more rapidly than any other nucleophiles. Initial studies assumed that they also follow Michael-1,4-addition reactions. Thus, the reaction of enzymatically generated dopaquinone with cysteine was assumed to produce 6-cysteinyldopa. However, careful examination of the structure of the product as well as quantitative product analysis revealed that the majority (about 3/4th) of the product is 5-cysteinyldopa, which is 1,6-addition product. Another isomer of 1,6-adduct, namely 2-cysteinyldopa is produced at about 14% level and the Michael-1,4-adduct (6-cysteinyldopa) is produced only at 1% level [1,33,34]. The reaction of *N*-acetylcysteine with *N*-acetyldopamine quinone also gives similar products [35]. The reason for this abnormal addition is not clear although various authors have proposed different hypothesis to account for this deviation. Thiol addition plays a crucial role in pheomelanin biosynthesis as cysteinyldopa and cysteinyldopamine formed by the condensation of cysteine with dopaquinone and dopaminequinone form the important precursors for pheomelanin biosynthesis in all animals. Even the thioether group of methionine is known to exhibit addition reaction with quinones that may parallel the reaction of thiols [36,37,38]. Thus, practically all-available biological nucleophiles can react with quinones producing various adducts. Apart from the reactions with these nucleophiles, certain if not all quinones are capable of undergoing isomerization to more reactive quinone methides.

## 3. Quinone Methides—General Consideration

Quinone methides are analogs of quinones with one of the carbonyl oxygen replaced with a benzylic methylene group. There are two types of quinone methides: *o*-quinone methide and *p*-quinone methide. Since only *p*-quinone methides are typically associated with catecholamines, the rest of the article will deal with the reactions of these novel intermediates and no aspect of *o*-quinone methide chemistry will be discussed.

**Figure 6:**
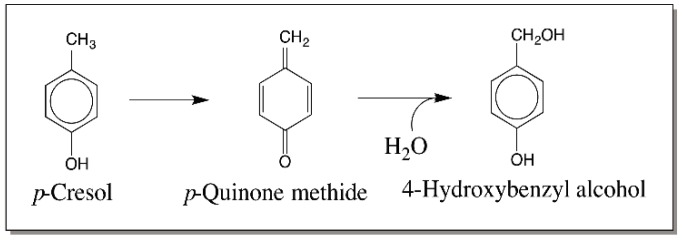
Rapid reaction of *p*-quinone methide. The simplest *p*-quinone methide arises from the two-electron oxidation of *p*-cresol. It is extremely unstable and reacts instantaneously with water forming the Michael-1,6-adduct, 4-hydroxybenzyl alcohol.

Quinone methides are extremely unstable and reactive. The alignment of dipoles in the functional groups of *p*-quinone methide lie in the same direction, which causes the oxygen to abstract a proton rapidly and force aromatization of the quinonoid nucleus. This results in the addition of even weaker nucleophiles at 6-position (Figure 6). Thus, the simplest *p*-quinone methide due to its extreme reactivity has not been isolated but has been characterized only as its Michael-1,6-adduct [39].

**Figure 7:**
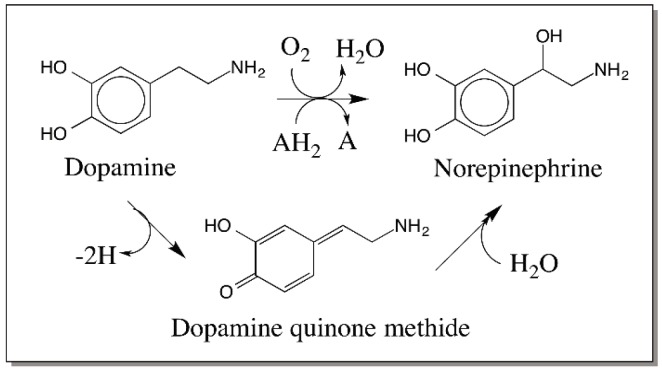
Mechanism of dopamine hydroxylation reaction. Initially, oxidation of dopamine to a quinone methide and hydration of the resultant quinone methide was proposed to be the route for the biosynthesis of norepinephrine. However, studies with labeled oxygen revealed that the hydroxylation is accompanied by the incorporation of one atom of molecular oxygen into dopamine by a specific dopamine-β-hydroxylase reaction.

**Figure 8:**
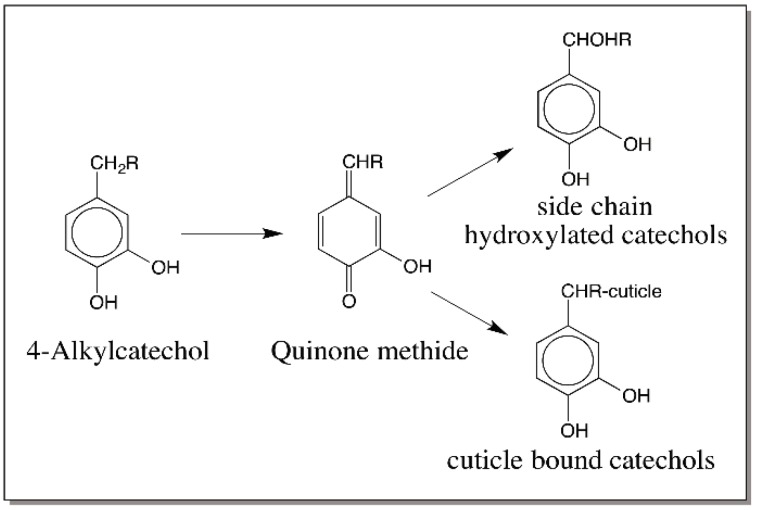
Initial observation on the quinone methide formation in insect cuticle. Incubation of 4-alkylcatechols with intact cuticle resulted in the covalent binding of catechols through their side chain to the cuticle and generation of side chain hydroxylated products.

In the case of catecholamine derivatives, as early as 1958, Witkop and others proposed that a similar route involving oxidation of dopamine to its *p*-quinone methide and subsequent hydration of the quinone methide to form the norepinephrine derivatives [40,41]. However, this novel route was disproved for the biosynthesis of norepinephrine and a direct enzymatic hydroxylation of dopamine side chain to give stereospecific norepinephrine was soon established [42]. Thus, dopamine β-hydroxylase catalyzes the stereospecific incorporation of one atom of molecular oxygen into the side chain of dopamine with the use of two reducing equivalents (Figure 7). Following this discovery, quinone methide involvement in catecholamine metabolism was completely abandoned until our group first reported a peculiar reaction of catecholamine derivatives with cuticular enzymes isolated from *Sarcophaga bullata* [43]. Incubation of intact cuticle isolated from the wandering stage of the *Sarcophaga bullata* larva, containing phenol oxidizing enzymes with various catecholamines produced side chain hydroxylated catechols and covalent binding of catechols through their side chain to the cuticle [43]. The reaction was accounted by the formation and subsequent reactions of quinone methide (Figure 8).

**Figure 9:**
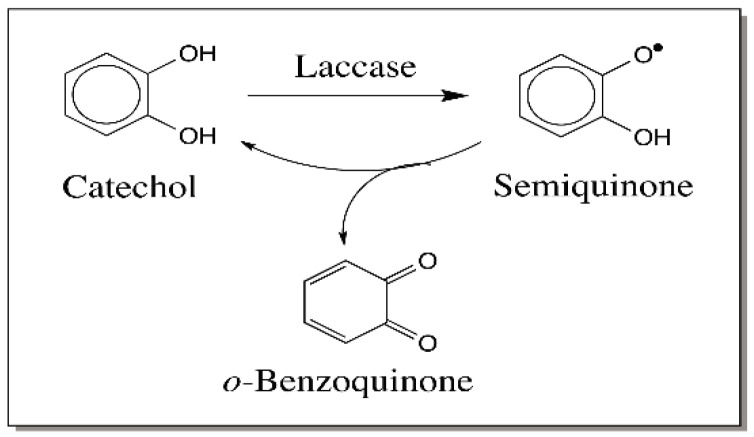
Laccase catalyzed oxidation of catechol. Laccase oxidizes catechols to the semiquinone. Two molecules of semiquinone undergo rapid disproportionation generating the starting material and quinone product.

**Figure 10:**
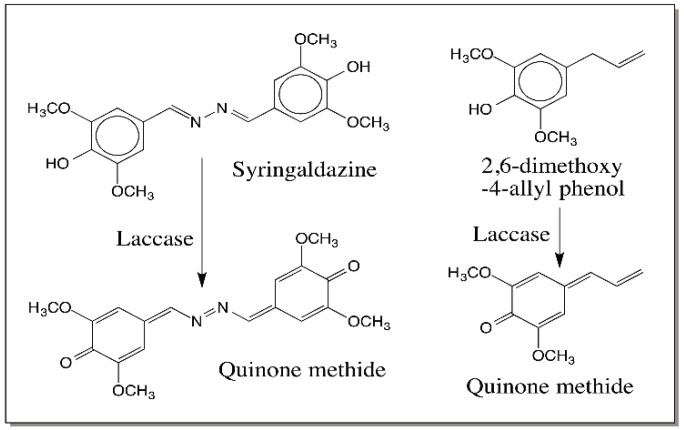
Laccase catalyzed quinone methide production from 4-alkyl phenols. Laccase produces quinone methide as the final product in some cases. For example, it oxidizes certain phenolic substrates such as syringaldazine and 2,6-dimethoxy-4-allylphenol to their corresponding semiquinones that undergo disproportionation to quinone methides and starting compounds.

Evidence that such an oxidation of 4-alkylphenols to quinone methide can occur easily comes from studies with laccase. Laccase form a heterogeneous group of phenoloxidases. They often catalyze the oxidation of *p*-diphenols to *p*-quinones. However, they also catalyze the oxidation of *o*-diphenols to *o*-quinones. Most laccases are associated with plants and fungi and are not present in higher animals. However, insects possess a large group of laccases for both cuticular pigmentation reaction and hardening reactions. The insect laccases specifically use *o*-diphenolic substrate such as dopamine. Laccases will oxidize their substrates to semiquinones through one electron oxidation [44]. However, the semiquinones, being unstable, react with one another undergoing disproportionation, which leads to the formation of the two-electron oxidation product, quinone and regeneration of the parent compound, catechol (Figure 9). Interestingly some but not all laccases will also oxidize certain phenols such as syringaldazine and 2,6-dimethoxy-4-allyl phenol to their corresponding quinone methides (Figure 10) [45]. Again the reaction occurs by disproportionation of the semiquinone to quinone methide and parent phenol. However, till now no one has demonstrated an enzyme that will oxidize 4-alkylcatechols to hydroxyquinone methides.

**Figure 11:**
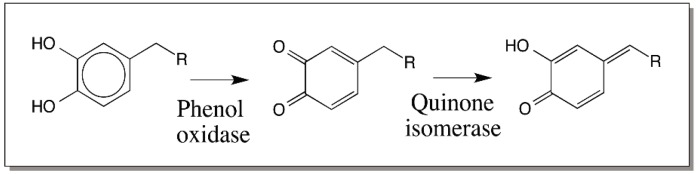
Mechanism of quinone methide production in insect cuticle. Quinone methide production from 4-alkylcatechols is accomplished in insect cuticle by a two-enzyme system consisting of phenoloxidase, which oxidizes the catechols to *o*-quinones and quinone isomerase that converts phenoloxidase generated 4-alkylquinones to *p*-quinone methides (R = alkyl substitution).

All our efforts to identify the enzyme that could generate quinone methides directly from 4-alkylcatechols by two electron oxidation ended up in vain. However, we were fortunate to identify a two-step route for this reaction [46,47]. Rather than direct generation of quinone methide from 4-alkylcatechols, we discovered a route involving initial oxidation of catechol to 4-alkylquinone by phenoloxidase and subsequent enzyme catalyzed isomerization of quinone to quinone methide by a quinone isomerase (Figure 11). Thus, our extensive efforts to search for the enzymes generating quinone methides resulted in the discovery of a new enzyme that isomerizes 4-alkyl quinones to hydroxyl quinone methides [47].

## 4. Predominance of Quinone to Quinone Methide Tautomerism in Simple Catechols

**Figure 12:**
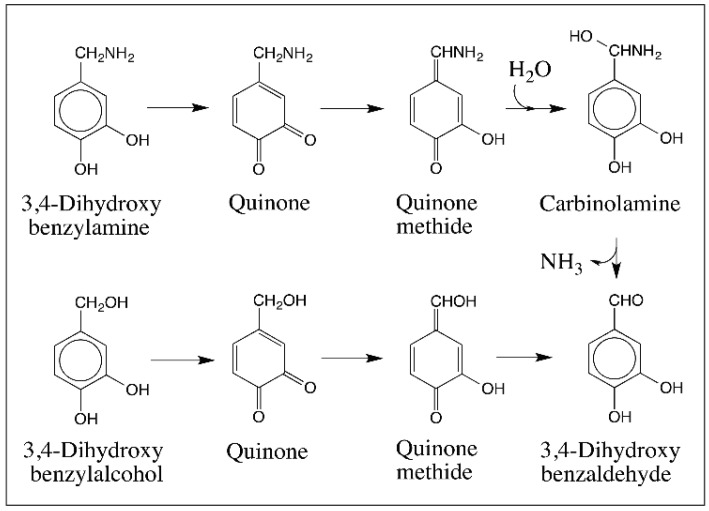
Oxidation chemistry of 3,4-dihydroxybenzylamine and 3,4-dihydroxybenzyl alcohol. 3,4-Dihydroxybenzylamine is easily oxidized to its quinone, which undergoes rapid nonenzymatic isomerization to quinone methide. Quinone methide reacts with water to form carbinolamine and then looses ammonia generating 3,4-dihydroxybenzaldehyde as the final product. A similar conversion of 3,4-dihydroxybenzyl alcohol to 3,4-dihydroxybenzaldehyde also occurs through a quinone methide intermediate.

Quinone isomerase is certainly needed for the conversion of *N*-acetyldopamine quinone to quinone methide [47] but a number of 4-substituted quinones exhibit spontaneous nonenzymatic isomerization reaction to quinone methides. Since these reactions are highly pertinent to eumelanogenesis, they are discussed in this section. Let us examine these reactions starting from C_6_–C_1_ compounds. Oxidation of 3,4-Dihydroxybenzylamine either chemically or enzymatically produces to its corresponding quinone, which is very unstable and isomerizes rapidly to the quinone methide. Michael-1,4-addition of water to quinone methide generates the carbinolamine intermediate (Figure 12). Substitution of hydroxyl group and amino group on the same carbon atom results in the loss of ammonia and generation of 3,4-dihydroxybenzaldehyde as the final product. [48]. 3,4-Dihydroxybenzyl alcohol also very much behaves the same way and produces 3,4-dihydroxybenzaldehyde as the final product, as shown in Figure 12 via another transient quinone methide [49].

**Figure 13:**
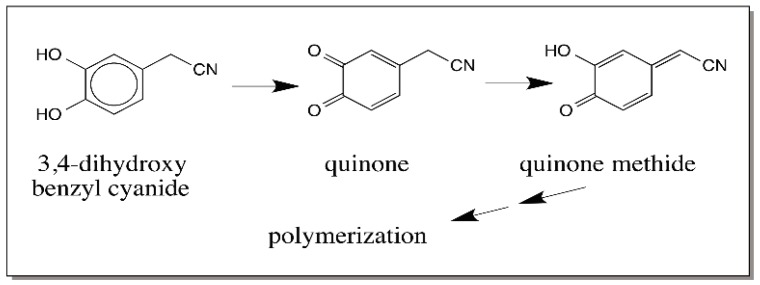
Oxidative transformation of 3,4-dihydroxybenzyl cyanide. 3,4-Dihydroxybenzyl cyanide upon oxidation to its quinone undergoes rapid isomerization to quinone methide, which readily polymerizes to uncharacterized products.

The next group of compounds that can exhibit quinone to quinone methide tautomerism is the C_6_–C_2_ series. 3,4-Dihydroxybenzyl cyanide upon oxidation readily produces the corresponding *o*-quinone. The benzylic carbon in this *o*-quinone is acidic due to the presence of electron withdrawing quinone nucleus and the cyanide group (Figure 13). As a result, it rapidly isomerizes to quinone methide. The quinone methide formed seems to undergo dimerization and perhaps other oligomerization reactions [50].

3,4-dihydroxyphenylglycine is the lower homologue of dopa. We synthesized this compound and studied its metabolic fate by oxidation reactions [51]. Tyrosinase readily oxidizes this compound to its quinone, which undergoes rapid decarboxylation to produce the same quinone methide as that produced by 3,4-dihydroxybenzylamine (shown in Figure 12). Thus, its oxidation also culminates in the same product viz., 3,4-dihydroxybenzaldehyde.

**Figure 14:**
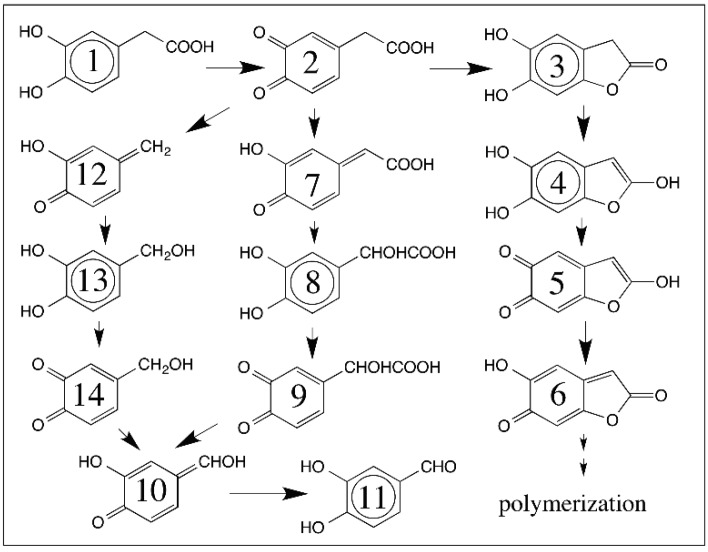
Multiple routes for the oxidative transformation of 3,4-dihydroxyphenylacetic acid. 3,4-Dihydroxyphenylacetic acid (**1**) is readily oxidized by tyrosinase to its quinone (**2**). This quinone exhibits three different reactions. It can undergo decarboxylation to the quinone methide (**12**), which reacts with water producing 3,4-dihydroxybenylalcohol (**13**). 3,4-dihydroxybenylalcohol is also oxidized by tyrosinase to its quinone (**14**) and nonenzymatically converted to 3,4-dihydroxybenzaldehyde (**11**) through quinone methide intermediate (**10**). Carboxymethyl-*o*-quinone (**2**) also isomerizes to quinone methide (**7**), which generates 3,4-dihydroxymandelic acid (**8**) by 1,6-addtion of water. Tyrosinase catalyzed oxidation of 3,4-dihydroxy mandelic acid produces the mandeloquinone (**9**), that is nonenzymatically decarboxylated to the same quinone methide (**10**) produced by 3,4-dihydroxybenzyl alcohol, thus yielding the same final product, 3,4-dihydroxy benzaldehyde (**11**). Finally, quinone (**2**) undergoes rapid intramolecular cyclization to form 2,5,6-trihydroxy benzofuran (**4**) via furanone (**3**). This furan is initially believed to form the quinone (**5**) before getting converted to quinone methide (**6**), and eventual polymerization.

3,4-Dihydroxyphenylacetic acid however, exhibits a more complex pattern of reactions which are summarized in Figure 14. A number of quinone to quinone methide tautomerism has been identified in this case [24,52,53,54]. Oxidation of 3,4-dihydroxyphenylacetic acid results in the formation of carboxymethyl-*o*-benzoquinone, which has three different fates. First it can undergo decarboxylation generating the simple hydroxyl *p*-quinone methide [53,54]. This quinone methide undergoes rapid Michael-1,6-addition reaction with water producing 3,4-dihydroxybenzyl alcohol (reaction **2** → **12** → **13**; Figure 14). The second reaction of carboxymethyl-*o*-benzoquinone is the conversion to isomeric quinone methide and rapid addition of water producing 3,4-dihydroxymandelic acid (reaction **2** → **7** → **8**; Figure 14) [24,54]. The mandelic acid derivative thus formed also undergoes further oxidation by tyrosinase. Initially we proposed a direct oxidative decarboxylation route to the formation of the quinone methide (compound **8** going directly to 10; Figure 14) [55]. However, it was subsequently demonstrated that mandeloquinone **9** is a compulsory intermediate, which undergoes rapid decarboxylation generating quinone methide as it is also a β,γ-unsaturated acid [54,56,57]. The dihydroxymandelate derivative in which the α-hydrogen is replaced with a methyl group also exhibits a similar reaction [58].

Finally, carboxymethyl-*o*-quinone exhibits a unique intramolecular cyclization reaction [24] that is similar to the intramolecular reaction observed with dopaquinone and dopaminequinone. This reaction produces 5,6-dihydroxybenzofuran-2-one (compound **3**; Figure 14). However, the furanone undergoes rapid oxidation producing a red colored compound, which was identified as the corresponding two-electron oxidation product—quinone methide (reactions **3** → **4** → **5** → **6**; Figure 14). At physiological pH, the formation of red colored quinone methide 6 could not be decisively identified [24]. However, with the use of fluorine substitution at the aromatic ring, we were able to show that the fluoroquinone looses fluoride ion during cyclization and produces the furanolactone, which is further oxidized to red colored quinone methide (**6**). The quinone (**5**) shown in Figure 14 is not formed at physiological pH, but the more stable yet highly reactive quinone methide (**6**) is generated during the oxidation [52].

**Figure 15:**
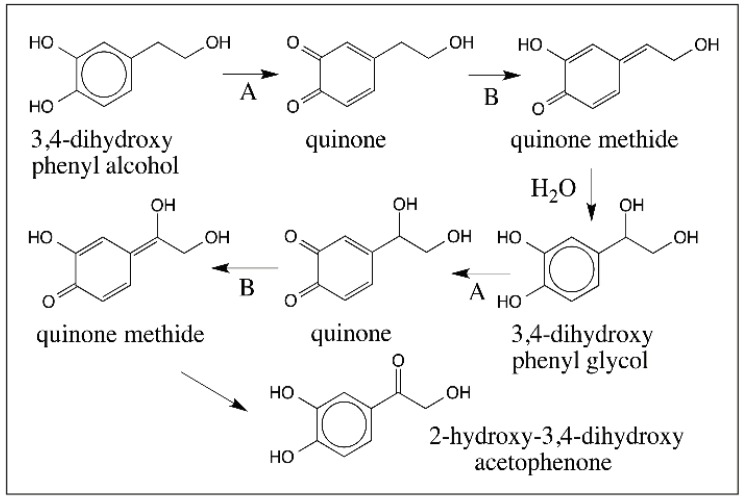
Oxidation of transformations of 3,4-dihydroxyphenethyl alcohol. Tyrosinase (**A**) catalyzes the oxidation of 3,4-dihydroxyphenethyl alcohol to its quinone, which can undergo either enzyme catalyzed isomerization or nonenzymatic isomerization (**B**) to its quinone methide. Water addition to this quinone methide and reoxidation of 3,4-dihydroxyphenyl glycol and yet another isomerization produces a transient quinone methide that readily yields 2-hydroxy-3,4-dihydroxy acetophenone as the final product.

Apart from these important compounds, two additional compounds—3,4-dihydroxyphenethyl alcohol as well as its side chain hydroxylated product, 3,4-dihydroxyphenyl glycol also exhibit quinone to quinone methide tautomerism as shown in Figure 15. The quinone isomerization could be enzyme catalyzed [59] as well as nonenzymatic reaction [54]. Thus, *o*-quinone formed from 3,4-dihydoxyphenethyl alcohol is converted by quinone isomerase to quinone methide, which rapidly reacts with water producing 3,4-dihydroxyphenyl glycol. Glycol undergoes further oxidation to quinone and isomerization to quinone methide. This quinone methide product of 3,4-dihydroxyphenyl glycol readily transforms by isomerization reaction to 2-hydroxy-3,4-dihydroxyacetophenone similar to the transformation of 3,4-dihydroxybenzyl alcohol quinone methide to 3,4-dihydroxybenzaldehyde as shown in Figure 12 [54,59].

**Figure 16:**
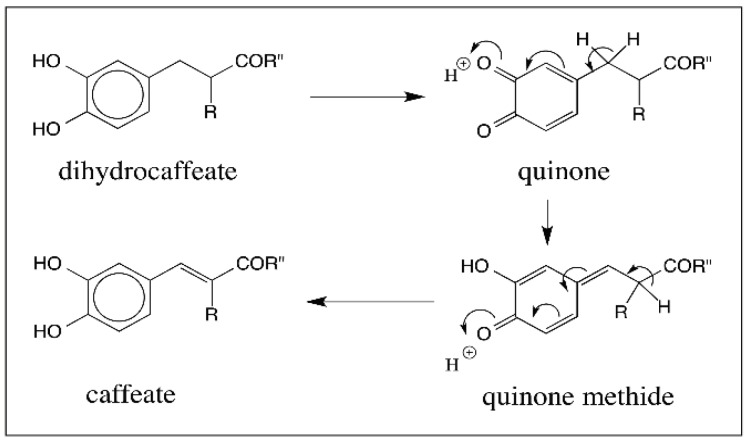
Oxidative transformation of dihydrocaffeate derivatives. Dihydrocaffeate derivatives (R” = NHCH_3_ or OCH_3_) upon oxidation produce the unstable quinones that exhibit rapid tautomerization to quinone methides. These quinone methides suffer another nonenzymatic isomerization producing more stable caffeic acid derivatives. This reaction also occurs with *N*-acetyldopa esters (R = NHCOCH_3_; R” = OCH_3_ or OCH_2_CH_3_).

Quinone to quinone methide tautomerism is much more prevalent in C_6_–C_3_ series, which is pertinent to melanin biosynthesis as dopa falls in this category. Even though dopamine and its derivatives belong to C_6_–C_2_ series, due to their participation in melanogenic process and their relation to dopa, their chemistry is considered in this section. Our group first showed that the quinones of dihydrocaffeic acid derivatives (both methylamide and methyl ester derivatives) exhibit facile tautomerization to quinone methide [25,60]. Oxidation of dihydrocaffeic acid derivatives readily produced the unstable quinones, which rapidly isomerized to quinone methide and then produced caffeic acid derivatives as the final products through yet another nonenzymatic isomerization reaction as shown in Figure 16. Subsequently, *N*-acetyldopa ethyl ester and *N*-acetyldopa methyl ester, possessing the same skeleton, were also shown to exhibit similar side chain desaturation reactions producing dehydro-*N*-acetyldopa esters [61,62].

Extensive kinetic studies carried out by Bolton and her associates [63,64] indicate that the rate of quinone isomerization to quinone methide heavily depends on the stability of the quinone methide. Extended π conjugation on the side chain carbon atom increased the stability of the quinone methide Thus, for example quinone methide formation from quinones of 4-propyl catechol, hydroxychavicol, and 4-cinnamoyl catechol increased dramatically with increased conjugation [63]. Bolton et al. [64] also examined the mechanism of isomerization of 4-propyl-*o*-quinone to its quinone methide and deduced that the isomerization reaction is a base catalyzed reaction with the abstraction of the proton at the quinone methide carbon by the base as the rate determining step of the reaction. Consistent with this finding they also observed a large kinetic isotope effect with the substitution of benzylic methylene protons with deuterium [64]. From the foregoing discussion, it is evident that quinone to quinone methide tautomerism plays a dominant role in the metabolic transformation of numerous catechols. The importance of quinone methide chemistry for melanin biosynthesis will be brought to light in the rest of this review.

## 5. Dopachrome Conversion and Quinone Methide Formation in Melanin Biochemistry

**Figure 17:**
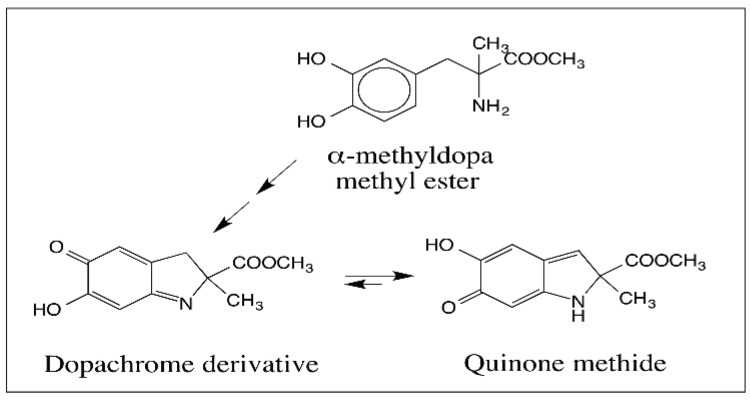
First demonstration of quinone methide production in a dopachrome derivative. α-Methyl dopa methyl ester upon oxidation produces its dopachrome derivative, which rapidly isomerizes to quinone methide under physiological conditions.

As early as 1980, Musson et al. [65] oxidized α-methyl dopa methyl ester by potassium ferricyanide and isolated a yellow colored solid whose structure was determined to be a quinone methide. However, its relation to melanin biosynthesis was not clarified until 1990 when we first reported the production of this quinone methide by isomerization of the dopachrome derived from α-methyl dopa methyl ester (Figure 17) [66]. Following our work, Prota and his associates also published evidence to support quinone methide from dopachrome derivatives [67,68]. Based on the facile isomerization of substituted dopachrome to a stable quinone methide [66], we postulated that a similar quinone methide should be formed during melanin biosynthesis as well. For the conversion of dopachrome to 5,6-dihydroxyindole, initially a route involving indolidine was proposed [69]. However, this route invokes electron donating properties for the C=N bond. However, the C=N bond is electron withdrawing in the C → N direction. As a consequence, it is easier to protonate the nitrogen. This will trigger electron flow around the ring causing the production of a transient quinone methide rather than the indolidine. Thus, quinone methide production is a more likely possibility. Once formed, the quinone methide can loose the carboxyl group as carbon dioxide or the α-proton to generate the 5,6-dihydroxyindole derivatives. Note that the quinone methide is a β,γ-unsaturated acid. Therefore, it can spontaneously undergo decarboxylation generating DHI. Indolidine however, is doubtful to exhibit this reaction. Although both indolidine and quinone methide can exhibit isomerization to DHICA. It should be pointed out here that various metal ions favor the production of DHI under nonenzymatic conditions [69]. Dopachrome generated at pH 6.5 also undergoes spontaneous decarboxylation to DHI while the same at pH 13 exhibits tautomerization to DHICA [70].

**Figure 18:**
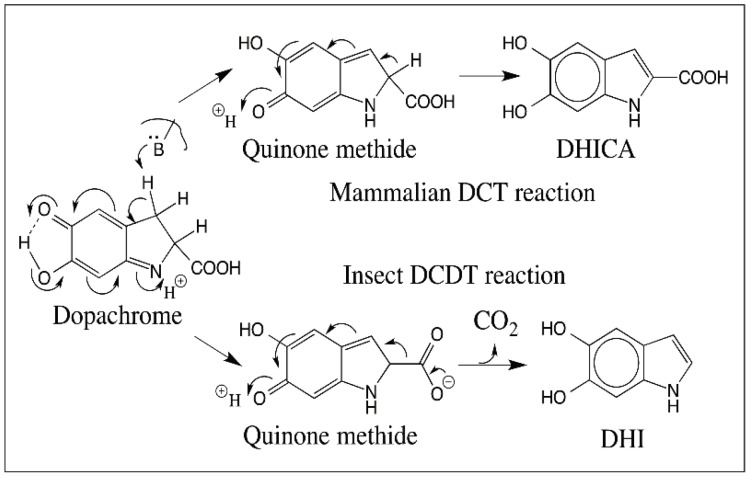
Reaction catalyzed by dopachrome converting enzymes. A base on the enzyme abstracts the β-proton on dopachrome causing isomerization of dopachrome to quinone methide. Quinone methide can readily exhibit another isomerization generating DHICA in the case of mammalian DCT. Since quinone methide is a β,γ-unsaturated acid, it can also readily exhibit decarboxylation. With insect DCDT, the reaction accompanies decarboxylation-coupled aromatization resulting in the formation of DHI.

Our group established concrete evidence for the participation of quinone methide in melanin biosynthesis in 1990 with the use of insect DCDT [19]. Insect enzyme exhibits wide substrate specificity and catalyzes the conversion of a number of l-dopachrome derivatives but does not touch their d-form [19]. It converts l-dopachrome to DHI. Similarly it converts α-methyldopachrome to 2-methyl-DHI, by decarboxylative rearrangement. However, with dopachrome methyl ester, which cannot undergo decarboxylation, it produces the isomeric product, 2-carboxymethyl DHI [19]. Finally it even acts on α-methyldopachrome methyl ester, which cannot undergo decarboxylation or deprotonation at the α-carbon producing the stable quinone methide [19]. Thus, dopachrome isomerase does not touch the α-proton or decarboxylate the carboxyl group, but removes the β-proton as the initial step leading to quinone methide production (Figure 18). Quinone methide undergoes either rapid decarboxylation to DHI with the insect enzyme and isomerization to DHICA with the mammalian enzyme (Figure 18).

## 6. Quinone Methide Intermediate in Other Catecholamine Reactions

**Figure 19:**
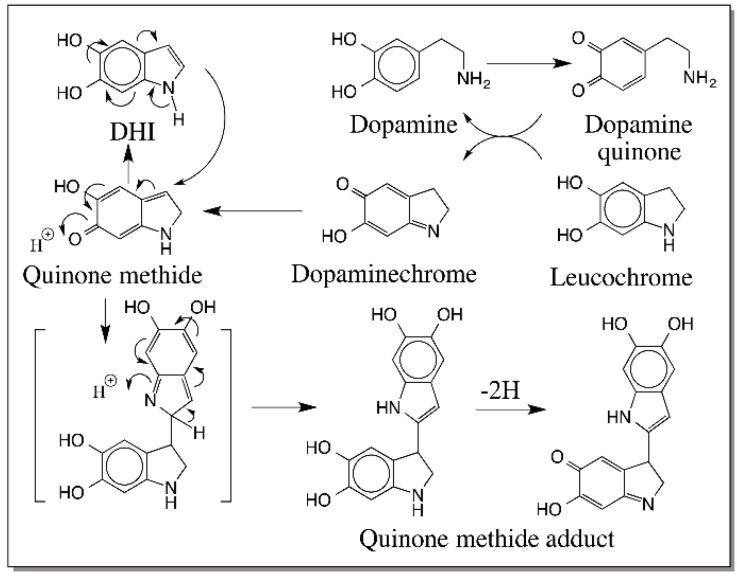
Formation of quinone methide adducts of dopamine during its oxidation. Dopamine quinone formed by the oxidation of dopamine undergoes intramolecular cyclization producing the leucochrome and then dopaminechrome. Isomerization of dopaminechrome to its quinone methide and aromatization of the catecholic ring produces DHI. A quinone methide adduct of DHI in its oxidative stage has been characterized from the reaction mixture.

Much like the same way dopa is converted to DHICA, dopamine is transformed into DHI by oxidative transformation [27]. Thus, dopaminequinone formed by the two-electron oxidation of dopamine undergoes rapid nonenzymatic intramolecular cyclization forming leucochrome, which is quickly oxidized by dopamine quinone to dopaminechrome (Figure 19). The conversion of dopaminechrome to DHI parallels the conversion of dopachrome to DHICA, thus obligating the formation of quinone methide intermediate. In this case, concrete evidence for the quinone methide intermediate comes from the product analysis studies. A quinone methide adduct of DHI at 2-postion was identified in the reaction mixture, the formation of which is shown in Figure 19 [27].

**Figure 20:**
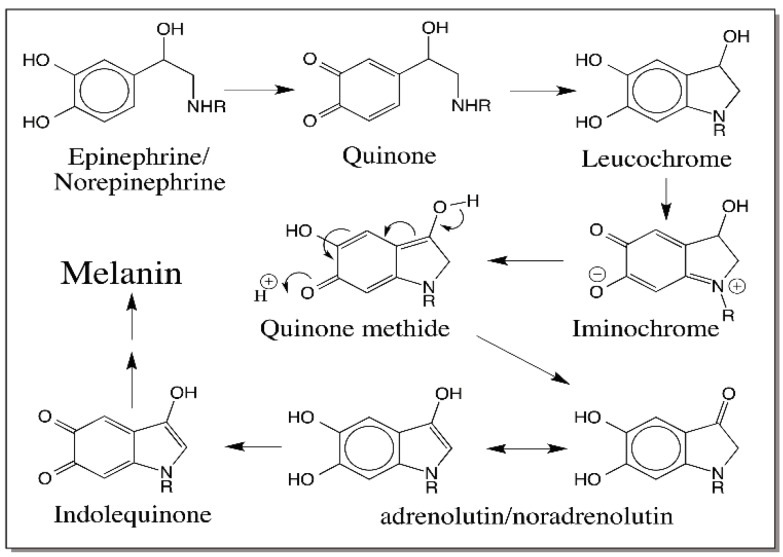
Oxidation of epinephrine and norepinephrine. Oxidation of epinephrine (R = methyl group) and norepinephrine (R = H) results in their corresponding quinones. These quinones also cyclize and get oxidized to form iminochromes. The iminochromes upon conversion to quinone methide easily isomerize and produce adrenolutin/noradrenolutin, which will also form melanin pigment eventually.

Oxidations of norepinephrine as well as epinephrine exhibit the quinone → leucochrome → iminochrome → indole transformations (Figure 20). The presence of hydroxyl group at 3-position will allow the quinone methide intermediate to rearrange and form the adrenolutin/noradrenolutin [71,72]. Norepinephrine quinone has been shown to isomerize to quinone methide and produce 2-hydroxy-3′,4′-dihydroxyacetophenone and a number of side chain oxidized products [72].

## 7. Reactivity of Dihydroxyindoles

**Figure 21:**
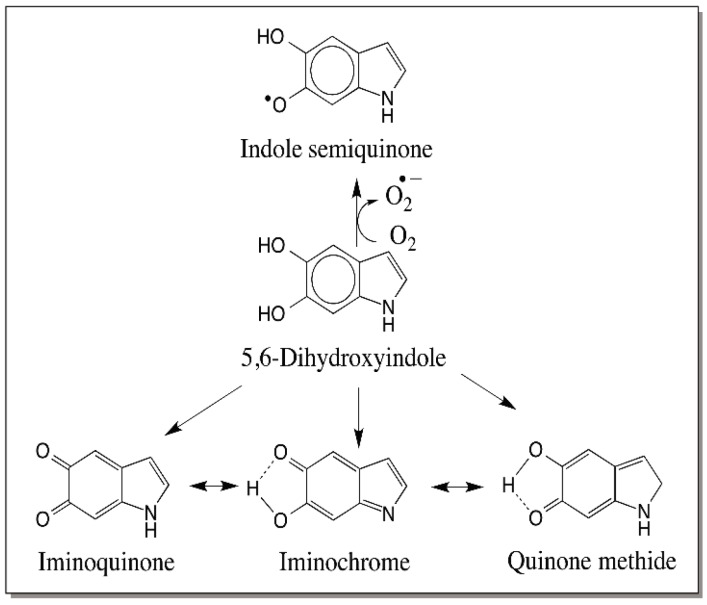
Possible reactive intermediates generated from DHI. DHI can undergo easily oxidation with molecular oxygen producing reactive semiquinones. In addition it is also susceptible to two-electron oxidation generating quinone, quinone methide and iminochrome.

A major difference between simple catechols and dihydroxyindoles is the presence of amino group attached to the catecholic ring in the latter compounds. This introduces extreme reactivity to dihydroxyindoles. People working with DHI for example are well aware of its extreme sensitivity to oxygen. DHI like its leucochrome precursor will also undergo rapid oxidation and produce the more stable quinone imine form, which will be in equilibrium with the isomeric quinone as well as the quinone methide. The indole quinone is less favored structure over the indolechrome or quinone methide, as both of these will give some stability due to internal hydrogen bonding (Figure 21). Thus, the two-electron oxidation of dihydroxyindole produce quinone, quinone imine and quinone methide isomers each one of which are capable of a plethora of reactions. Among the two electron oxidation products, the iminochrome form will be less reactive compared to the quinone methide form. A good example for this is the reactivity of dopachrome and its quinone methide isomer. The quinone methide is very reactive compared to dopachrome in its iminochrome form. Thus, quinone methide form of two electron oxidation product of DHI will have a dominant effect on the course of the reaction because quinone methide can undergo easy Michael-1,6-addition reaction. Molecular oxygen as well as other oxidants such as dopachrome and dopaquinone can also cause the oxidation of DHI. Oxidases including peroxidase and tyrosinase will also catalyze the oxidation. Both one electron oxidation and two-electron oxidation are possible for DHI (Figure 21). Amino catechols as well as trihydroxyphenols such as 6-hydroxydopa derivatives exhibit easy one electron oxidation to produce semiquinone radicals both by peroxidase catalyzed reaction and oxidation with molecular oxygen. Further coupling of free radicals will produce a number of oligomeric products. At present a separate enzyme catalyzing the oxidation of DHI seems to be doubtful, as there are a number of reactants that can easily oxidize this compound. However, the same cannot be said for DHICA. The presence of carboxyl group in DHICA stabilizes the amino group by hydrogen bonding and prevents easy oxidation of this compound. Thus, DHICA is more stable that DHI.

**Figure 22:**
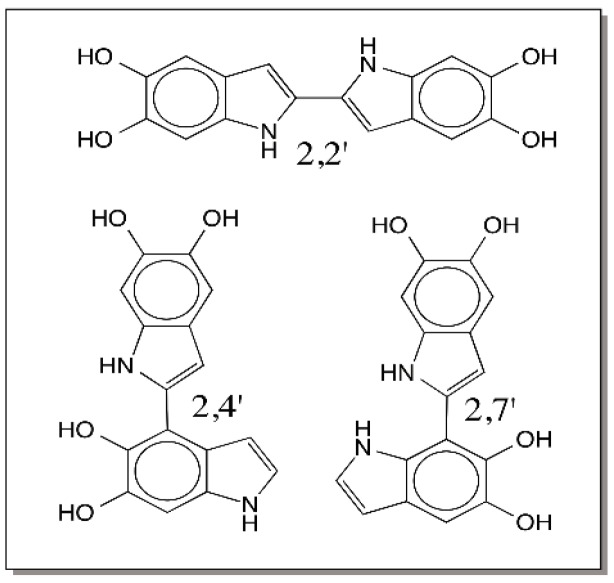
Structure of DHI dimers. Oxidative polymerization of DHI yields 2,2′, 2,4′ and 2,7′ coupled DHI dimers as the major products.

The reactive species formed from dihydroxyindoles also react with each other undergoing self-polymerization thereby producing melanin pigment. Of the seven possible positions, hydroxyl groups occupy two positions and one position is used up by NH group in the ring. This leaves position 2 and 3 on the indole ring and position 4 and 7 on the catechol ring for polymerization reactions. Quinones typically undergo Michael-1,4-addition reactions; but these two positions are not available in the quinone structure. There is some possibility of reaction occurring *ortho* to the quinone carbonyl group. In comparison, the quinone methide carbon should be extremely reactive for addition reactions. This will produce 3-substituted indoles and regeneration of the aromatic ring. Evidence for the quinone methide reactivity comes from mass spectral studies of the oxidative transformation of dopamine. Kroesche and Peter [73] observed that during the oligomerization reaction of DHI originating from dopamine, there was an incorporation of one atom of oxygen into the indole units. Such incorporation can be accounted by the reaction of quinone methide with water to generate 3-hydroxy 5,6-dihydroxyindole units. Al-Kazwini et al. [74,75] also witnessed the quinone methide formation during both two-electron and one-electron oxidation of DHI. However, the majority of the dimeric products identified during the oxidative coupling of DHI seem to be couple at the 2-position [76,77,78,79,80]. Figure 22, for example, shows the structure of the three major products characterized from the DHI reaction mixture.

Interestingly, the oxidation products of some of these dimers employ quinone methide reactivity [79,80,81]. A 2,3′ coupled tetramer was identified during the oxidative dimerization of the 2,4′ dimer [80]. Two different dimeric products of quinone methide coupled products (2,3′ and 3,3′) have been characterized during the oxidative dimerization of 2,7′ dimer [80].

**Figure 23:**
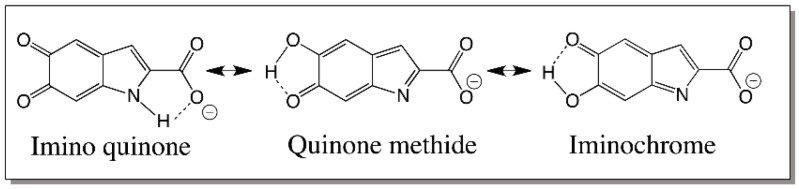
Two-electron oxidation products of DHICA. Two electron oxidation of DHICA will produce imino quinone, quinone methide and iminochrome products.

**Figure 24:**
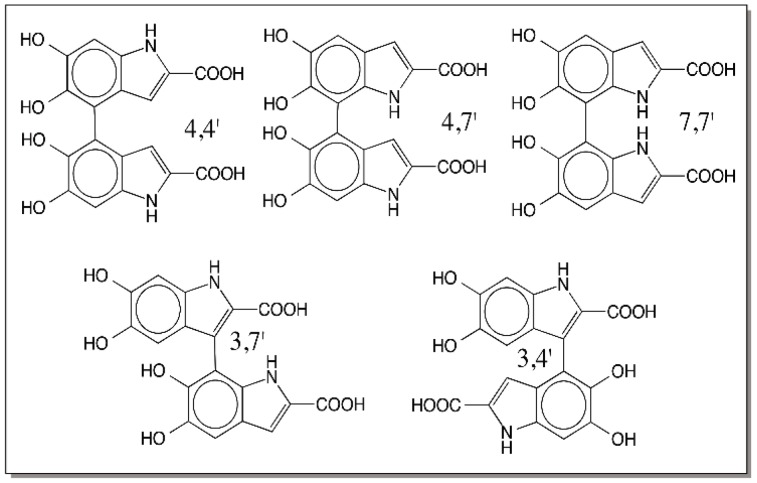
Structure of DHICA dimers. Oxidative polymerization of DHIDA yields 4,4′, 4,7′ and 7,7′ coupled DHICA dimers as the major products and 3,4′ and 3,7′ coupled dimers as the minor products.

Two-electron oxidation of DHICA can produce three different isomeric quinonoid products. These are quinone imine, quinone methide and iminochrome forms which are depicted in Figure 23. All of which are capable of having hydrogen-bonding stabilization as shown in the same figure. Of which iminochrome form should be theoretically more stable than the other two forms. Iminochrome gets its stabilization by the presence of opposing dipole movements by the *para* positioned C=O and C=N bonds. On the other hand, both quinone and quinone methide should be more reactive as their quinonoid group is poised readily for addition reactions. There are only three possible positions to exhibit reactivity in these molecules that can cause re-aromatization. The favorable 2-postion that is free in DHI is occupied in DHICA by a carboxyl group thus preventing any coupling reaction at this position. Thus, only 3-, 4-, and 7-positions are available for coupling in this molecule. Again, the reactive position should be theoretically the 3-position, but so far only products coupled at 4- and 7-position have been identified as major products and 3 coupled products have been identified as minor products (Figure 24) [82,83,84].

## 8. Comparative Oxidation Chemistry of 1,2-Dehydro-*N*-acyldopamines and Dihydroxyindoles

In order to understand the significance of quinone methide reactivity for eumelanogenesis, it is important to consider the reactivities of a sister group of compounds called 1,2-dehydro-*N*-acyl dopamine derivatives. The simplest compound in this group is 1,2-dehydro-*N*-acetyldopamine (dehydro NADA). It is biosynthesized in a vast majority of insects for cuticular hardening process also known as sclerotization [3,30,31,32]. The reactive intermediates generated during sclerotization form adducts with cuticular proteins and chitin backbone that results in a hardened cuticle. This process is very similar to melanin biosynthesis with the exception that the reactive intermediates generated in cuticle are better suited to exhibit external reactions while the reactive intermediates of melanogenic pathway are poised for internal polymerization reaction. The reactions associated with both sclerotization and melanization exhibit remarkable similarity between each other [32]. The catecholamine derivatives *N*-acetyldopamine and *N*-β-alanyldopamine are precursor for sclerotization while dopa and dopamine are precursor for melanization, the major difference between them being the protection of amino group by acylation, which prevents internal reactivity in the case of sclerotizing precursors. Tyrosinase initiates melanogenesis by oxidizing the catechols to quinones, while the related enzyme, phenoloxidase initiates sclerotization doing the same oxidation. *N*-acyldopamine quinones exhibit external reactivity, while dopa/dopamine quinones exhibit internal reactivity. *N*-acyldopamine quinones are converted to quinone methide derivatives by quinone isomerase [46,47]. Dopachrome tautomerases—DCT and DCDT—convert dopachrome to DHICA and DHI respectively in melanogenesis [12,13,14,15,16,17,18,19]. A separate quinone methide isomerase causes the isomerization of *N*-acyldopamine quinone methides to 1,2-dehydro-*N*-acyldopamines [85,86,87]. Thus, the double bond is introduced in the side chain of dopa derivatives by extremely similar enzymatic reactions in both the pathways.

**Figure 25:**
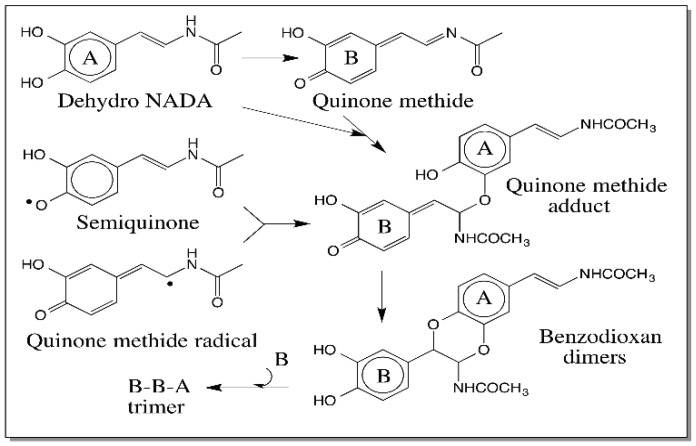
Oxidative transformations of dehydro NADA. Dehydro NADA upon oxidation produces directly a highly reactive quinone methide, which crosslinks proteins and chitin (not shown in figure). It also reacts with the starting compound forming a benzodioxan dimer. The accumulated dimer reacts with another molecule of quinone methide forming trimer. Oligomers up to hexamer have been characterized. One-electron oxidation of dehydro NADA generates the semiquinone, which is in equilibrium with the quinone methide radical. Radical coupling in this case produces the same dimer but not the oligomers.

Extensive studies carried out on the oxidation chemistry of 1,2-dehydro-*N*-acetyldopamine (dehydro NADA) paved way to understand the key role played by quinone methides in the sclerotization process [88,89,90,91]. Dehydro NADA upon two electron oxidation produces a transient quinone methide and not the conventional quinone product. This quinone methide forms adduct with proteins and chitin generating adducts and crosslinks necessary for strengthening and hardening the cuticle. In addition, it also forms adduct with the parent catechols generating benzodioxan type compounds as shown in Figure 25. Moreover, the quinone methide also reacts with the catecholic group of dimeric products generating trimers, then tetramers, and a number of oligomers [91].

**Figure 26:**
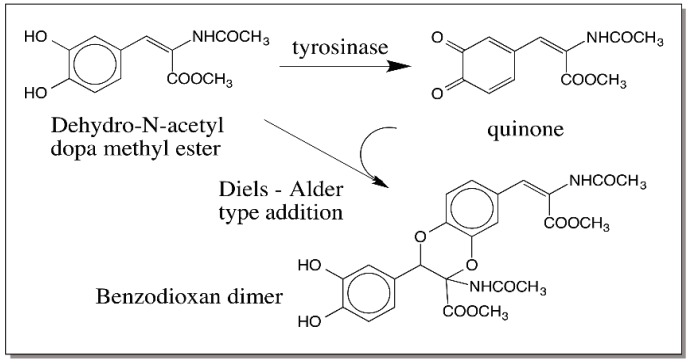
Oxidative dimerization of 1,2-dehydro-*N*-acetyldopa methyl ester. Oxidation of 1,2-Dehydro-*N*-acetyldopa methyl ester by tyrosinase results in the production of conventional quinone product and not the quinone methide analog. This quinone also dimerizes and produces the same benzodioxan type adduct as that produced by dehydro NADA but by an ionic Diels–Alder type reaction.

Interestingly even the one-electron oxidation product forms the same dimeric adduct through a radical coupling reaction. Since radical coupling results in the precipitous loss of semiquinone radicals, in this case, only dimers are formed as shown in Figure 25 [91]. This kind of dimerization reaction is manifested by a number of dehydro dopyl derivatives [26,88,89,90,91,92,93,94]. The quinone to quinone methide tautomerism is very much in favor of quinone methides in the case of dehydro NADA [90] as well as 1,2-dehydro-*N*-acetyldopa [26]. However, with 1,2-dehydro-*N*-acetyldopa methyl ester, quinone product is slightly more stable than the quinone methide [94]. However, even in this case, the quinone exhibits an ionic Diels–Alder type addition forming the same benzodioxan dimeric products (Figure 26) [94]. Thus, in dehydro dopa derivatives both the side chain positions seem to be extremely reactive. Oxidized DHI and DHICA should also exhibit the same kind of additions.

## 9. Oligomerization of DHI and DHICA and the Final Eumelanogenic Pathway

Several authors have demonstrated the facile oligomerization of both DHI and DHICA during eumelanogenesis. As stated earlier, a number of dimeric structures of DHI and DHICA have been characterized [73,74,75,76,77,78]. A mixed dimer of DHI and DHICA has also been identified [95]. Trimeric [77,83] as well as tetrameric products [79,80,81] of DHI and DHICA have also been characterized. Using mass spectral analysis, Kroesche and Peter [73] identified oligomers of up to eleven units of DHI. Studies with matrix-assisted laser desorption/ionization mass spectrometry have revealed the production of dimeric to heptomeric linear oligomers of DHICA coupled at 4,7-positions [96]. During a study of tyrosinase catalyzed oxidation of tyrosine, Bertazzo et al. [97] witnessed the production of not only DHI oligomers as well as DHICA oligomers, but also mixed DHI-DHICA oligomers. Furthermore, they were able to observe the production of DHI oligomers up to nanomer. Structural analyses of several of these compounds are extremely difficult due to nonavailability of sufficient materials and the extreme instability of oligomeric products. As pointed out in Section 7 on the reactivities of dihydroxyindoles, a number of these adduct formation involves quinone methide participation. Thus, eumelanogenic pathway has emerged to be a more complex biochemical pathway than what is described in Figure 1.

**Figure 27:**
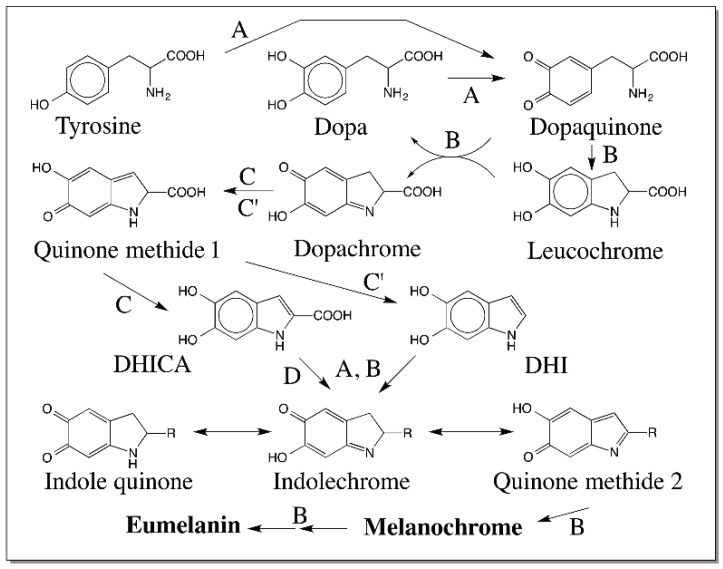
Modified Raper–Mason Pathway for eumelanin biosynthesis. Tyrosinase (**A**) converts tyrosine to dopaquinone and oxidizes dopa to dopaquinone. External addition of cysteine to dopaquinone and the oxidative polymerization of cysteinyldopa result in the production of pheomelanin pigments (not shown in Figure). Intramolecular cyclization of dopaquinone produces leucochrome, which is further oxidized to dopachrome by nonenzymatic reactions (**B**). Dopachrome is isomerized to transient quinone methide (1) that is converted to either DHICA by mammalian DCT (**C**) or DHI by insect DCDT (**C′**). Oxidation of these two indoles by tyrosinase or nonenzymatic reactions or by DHICA oxidase (**D**) produces among other reactive species, quinone methides, which will have a major role in eventual polymerization of these monomeric compounds (R = H for DHI derivative; and COOH for DHICA derivative).

Based on the foregoing discussion and taking into consideration of the key roles played by quinone methide intermediates during oxidative transformation of many catecholamine derivatives, one can formulate a modified Raper–Mason pathway for eumelanin biosynthesis as shown in Figure 27. Tyrosinase has been shown to convert tyrosine to dopaquinone directly and not through the intermediacy of dopa [98]. Tyrosinase can also oxidize dopa to dopaquinone. The intramolecular cyclization of dopaquinone to leucochrome and its further oxidation by dopaquinone produces dopachrome and dopa. Thus, dopa is generated only nonenzymatically by an indirect route. While the above reactions corroborate the importance of quinones, the subsequent reactions attest the key role of quinone methides in eumelanogenic pathway. Two quinone methide intermediates—one at the stage of dopachrome conversion and the other at the stage of dihydroxyindole oxidation—are shown. The monomeric units of indole quinone, iminochrome and quinone methide thus formed undergo oligomerization and eventual polymerization. The dimers, trimers, tetramers and other oligomers upon oxidation will form reactive quinone methide derivatives and exhibit further polymerization as demonstrated by the pioneering work of the Italian group [76,77,78,79,80,95,96]. Thus, quinone methides seem to play a dominant role not only in the middle part but also in the later part of eumelanin biosynthesis. As indicated in Section 2, the reactions of quinones are typically manifested through the ring additions, where as the reactions of quinone methides are centered on the side chain additions. A combination of all these reactivities makes the eumelanin biosynthesis very intriguing and complex.

## 10. Conclusions

In spite of many decades of intensive research, several aspects of structure, reaction course and biochemical mechanism of eumelanin pigment still remains unresolved. One such aspect is the production and use of reactive quinone methide intermediates. Ever since our group first demonstrated the involvement of quinone methide in the oxidation chemistry of α-methyl dopa methyl ester, a number of quinone methide derivatives have been shown to be reactive intermediates during the metabolic transformation of a plethora of catecholamine compounds as summarized in this review. Quinone methides play crucial role not only in melanin biosynthesis but also in insect cuticular sclerotization process that is very much related to melanin biosynthesis. To date, four enzymes that are solely involved in quinone methide biochemistry have been identified: mammalian DCT that produces DHICA from dopachrome; insect DCDT that produces DHI from dopachrome; quinone isomerase that converts *N*-acyldopamine to their corresponding quinone methides; and quinone methide isomerase that isomerizes *N*-acyldopamine quinone methide to dehydro-*N*-acyldopamine. Due to high reactivity and extreme instability quinone methide formation and utilization in several biological processes including melanogenesis remains still to be under appreciated. Only future studies can shed more light on the many aspects of quinone methide reactivity in melanogenesis process.

## Abbreviations

DHI	5,6-dihydroxyindole
DHICA	5,6-dihydroxyindole-2-carboxylic acid
Dehydro NADA	1,2-dehydro-*N*-acetyldopamine
DCT	Dopachrome tautomerase
DCDT	Dopachrome decarboxylase/tautomerase

## References

[1] Prota G. (1992). Melanins and Melanogenesis.

[2] Hill H.Z. (1992). The function of melanin or six blind people examine an elephant. BioEssays.

[3] Sugumaran M. (2002). Comparative biochemistry of eumelanogenesis and the protective roles of phenoloxidase and melanin in insects. Pigment Cell Res..

[4] Ito S. (2003). A chemist’s view of melanogenesis. Pigment Cell Res..

[5] Hearing V.J., Tsukamoto K. (1991). Enzymatic control of pigmentation in mammals. FASEB J..

[6] Raper H.S. (1926). The tyrosinase-tyrosine reaction. Production of l-3,4-dihydroxyphenylalanine from tyrosine. Biochem. J..

[7] Raper H.S. (1928). The aerobic oxidases. Physiol. Rev..

[8] Raper H.S. (1938). Some problems of tyrosine metabolism. J. Chem. Soc..

[9] Mason H.S. (1948). The chemistry of melanin. III. Mechanism of the oxidation of dihydroxyphenylalanine by tyrosinase. J. Biol. Chem..

[10] Mason H.S. (1955). Comparative biochemistry of the phenolase complex. Adv. Enzymol..

[11] Korner A.M., Pawelek J. (1980). Dopachrome conversion factor: A possible control point in melanin biosynthesis. J. Investig. Dermatol..

[12] Pawelek J. (1990). Dopachrome conversion factor functions as an isomerase. Biochem. Biophys. Res. Commun..

[13] Aroca P., Solano F., Garcia-Borron J., Lozano J. (1990). A new spectrophotometric assay for dopachrome tautomerase. J. Biochem. Biophys. Methods.

[14] Aroca P., Garcia-Borron J., Solano F., Lozano J. (1990). Regulation of mammalian melanogenesis I: Partial purification and characterization of a dopachrome converting factor: Dopachrome tautomerase. Biochim. Biophys. Acta.

[15] Pawelek J.M. (1991). After dopachrome?. Pigment Cell Res..

[16] Tsukamoto K., Jackson I.J., Urabe K., Montague P.M., Hearing V. (1992). A second tyrosinase related protein, TRP-2, is a melanogenic enzyme termed DOPAchrome tautomerase. EMBO J..

[17] Aso Y., Kramer K.J., Hopkins T.L., Whetzel S.Z. (1984). Properties of tyrosinase and dopa quinone imine conversion factor from pharate pupal cuticle of *Manduca sexta*. Insect Biochem..

[18] Aso Y., Imamura Y., Yamasaki N. (1989). Further studies on dopa quinone imine conversion factor from the cuticles of *Manduca sexta*. Insect Biochem..

[19] Sugumaran M., Semensi V. (1991). Quinone methides as new intermediates of melanin biosynthesis. J. Biol. Chem..

[20] Jimenez-Cervantes C., Solano F., Kobayashi T., Urabe K., Hearing V.J., Lozano J.A., Garcia-Borron J.C. (1994). A new enzymatic function in the melanogenic pathway. The 5,6-dihydroxyindole-2-carboxylic acid oxidase activity of tyrosinase-related protein-1 (TRP1). J. Biol. Chem..

[21] Kobayashi T., Urabe K., Winder A., Jimenez-Cervantes C., Imokawa G., Brewington T., Solano F., Garcia-Borron J.C., Hearing V.J. (1994). Tyrosinase-related protein-1 (TRP1) functions as DHICA oxidase in melanin biosynthesis. EMBO J..

[22] Jane S.M., Mu D., Wemmer D., Smith A.I., Kaur S., Malby D., Burlingame A.I., Klinman J.P. (1990). A new redox cofactor in eukaryotic enzymes: 6-Hydroxydopa at the active site of bovine amine oxidase. Science.

[23] Wang S.X., Mure M., Medzihradszky K.F., Burlingame A.I., Brown D.E., Dooley D.M., Smith A.J., Kagan M., Klinman J.P. (2006). A crosslinked cofactor in lysyl oxidase: Redox function for amino acid side chains. Science.

[24] Sugumaran M., Semensi V., Dali H., Mitchell W. (1989). Novel oxidative transformations of 3,4-dihydroxy phenylacetic acid to 2,5,6-trihydroxybenzofuran and 3,4-dihydroxymandelic acid catalyzed by mushroom tyrosinase. Bioorg. Chem..

[25] Sugumaran M., Dali H., Kundzicz H., Semensi V. (1989). Unusual intramolecular cyclization and side chain desaturation of carboxyethyl-*o*-benzoquinone derivatives. Bioorg. Chem..

[26] Abebe A., Kuang Q.F., Evans J., Sugumaran M. (2013). Mass spectrometric studies shed light on unusual oxidative transformations of 1,2-dehydro-N-acetyldopa. Rapid Commun. Mass Spectrom..

[27] Zhang F., Dryhurst G. (1993). Oxidation chemistry of Dopamine: Possible insights into the age-dependent loss of dopaminergic nigrostriatal neurons. Bioorg. Chem..

[28] Blank C.L., Kissinger P.T., Adams R.N. (1972). 5,6-Dihydroxyindole formation from oxidized 6-hydoxy dopamine. Eur. J. Pharmacol..

[29] Huang X., Xu R., Hawley M.D., Kramer K.J. (1997). Model insect cuticle sclerotization: Reactions of catecholamine quinones with the nitrogen-centered nucleophiles imidazole and *N*-acetyldopamine. Bioorg. Chem..

[30] Sugumaran M. (2010). Chemistry of cuticular sclerotization. Adv. Insect Physiol..

[31] Sugumaran M. (2009). Complexities of cuticular pigmentation in insects. Pigment Cell Melanoma Res..

[32] Sugumaran M. (1991). Molecular mechanisms for mammalian melanogenesis—Comparison with insect cuticular sclerotization. FEBS Lett..

[33] Ito S., Prota G. (1977). A facile one step synthesis of cysteinyldopas using mushroom tyrosinase. Experientia.

[34] Kato T., Ito S., Fujita K. (1986). Tyrosinase-catalyzed binding of 3,4-dihydroxyphenylalanine with proteins through the sulfhydryl group. Biochim. Biophys. Acta.

[35] Sugumaran M., Dali H., Semensi V. (1989). Chemical and cuticular phenoloxidase mediated synthesis of cysteinyl catechol adducts. Arch. Insect Biochem. Physiol..

[36] Vithayathil P.J., Murthy G.S. (1972). New reaction of *o*-benzoquinone at the thioether group of methionine. Nature.

[37] Vithayathil P.J., Gupta M.N. (1981). Reaction of methionine with some biologically important *o*-quinones. Ind. J. Biochem. Biophys..

[38] Sugumaran M., Nelson E. (1998). Model sclerotization Studies: 4. Generation of *N*-acetylmethionyl catechol adducts during tyrosinase catalyzed oxidation of catechols in the presence of *N*-acetylmethionine. Arch. Insect Biochem. Physiol..

[39] Cunane L.M., Chen Z.-W., Shamala N., Scott Mathews F., Cronin C.N., McIntire W.S. (2000). Structures of the flavocytochrome *p*-cresol methylhydroxylase and its enzyme-substrate complex: Gated entry and proton relays support the proposed catalytic mechanism. J. Mol. Biol..

[40] Senoh S., Witkop B. (1959). Non-enzymatic conversions of dopamine to norepinephrine and trihydroxyphenethylamines. J. Am. Chem. Soc..

[41] Senoh S., Creveling C.R., Udenfriend S., Witkop B. (1959). Chemical, enzymatic and metabolic studies on the mechanism of oxidation of dopamine. J. Am. Chem. Soc..

[42] Kaufman S., Bridgers W.F., Eisenberg F., Friedman S. (1962). The source of oxygen in the phenylalanine hydroxylase and the dopamine-β-hydroxylase catalyzed reactions. Biochem. Biophys. Res. Commum..

[43] Sugumaran M., Lipke H. (1983). Quinone methide formation from 4-alkylcatechols. A novel reaction catalyzed by cuticular polyphenoloxidase. FEBS Lett..

[44] Nakamura T. (1960). On the process of enzymatic oxidation of hydroquinone. Biochem. Biophys. Res. Commun..

[45] Sugumaran M., Bolton J.L. (1998). Laccase—And not tyrosinase—Is the enzyme responsible for quinone methide production from 2,6-dimethoxy-4-allyl phenol. Arch. Biochem. Biophys..

[46] Saul S.J., Sugumaran M. (1989). *o*-Quinone: Quinone methide isomerase—A novel enzyme which prevents the destruction of self matter by phenoloxidase generated quinones during immune response in insects. FEBS Lett..

[47] Saul S.J., Sugumaran M. (1990). 4-Alkyl-*o*-quinone/2-hydroxy-*p*-quinone methide isomerase from the larvae hemolymph of *Sarcophaga bullata*. I. Purification and characterization of enzyme catalyzed reaction. J. Biol. Chem..

[48] Sugumaran M. (1995). Oxidation of 3,4-dihydroxybenzylamine affords 3,4-dihydroxybenzaldehyde via the quinone methide intermediate. Pigment Cell Res..

[49] Sugumaran M., Semensi V., Dali H., Nellaiappan K. (1991). Oxidation of 3,4-dihydroxybenzyl alcohol: A sclerotizing precursor for cockroach ootheca. Arch. Insect Biochem. Physiol..

[50] Cooksey C.J., Garrett P.J., Land E.J., Ramsden C.A., Riley P.A. (1998). Tyrosinase kinetics: Failure of the auto-activation mechanism of monohydric phenol oxidation by rapid formation of a quinomethane intermediate. Biochem. J..

[51] Sugumaran M., Tan S., Sun H.L. (1996). Tyrosinase catalyzed oxidation of 3,4-dihydroxyphenylglycine. Arch. Biochem. Biophys..

[52] Sugumaran M., Duggaraju R., Jayachandran K., Kirk K. (1999). Formation of a new quinone methide intermediate during the oxidative transformation of 3,4-dihydroxyphenylacetic acids: Implications for eumelanin biosynthesis. Arch. Biochem. Biophys..

[53] Mefford I.N., Kincl L., Dykstra K.H., Simpson J.T., Markey S.P., Dietz S., Wightman R.M. (1996). Facile oxidative decarboxylation of 3,4-dihydroxyphenylacetic acid catalyzed by copper and manganese ions. Biochim. Biophys. Acta.

[54] Ito S., Yamanaka Y., Wakamatsu K. (2016). The metabolic fate of *ortho*-quinones derived from catecholamine metabolites. Int. J. Mol. Sci..

[55] Sugumaran M. (1985). Tyrosinase catalyzes an unusual oxidative decarboxylation of 3,4-dihydroxymandelate. Biochemistry.

[56] Cabanes J., Sanchez-Ferrer A., Bru R., Garcia-Carmona F. (1988). Chemical and enzymic oxidation by tyrosinase of 3,4-dihydroxymandelate. Biochem. J..

[57] Sugumaran M., Dali H., Semensi V. (1992). Mechanistic studies on tyrosinase catalyzed oxidative decarboxylation of 3,4-dihydroxymandelic acid. Biochem. J..

[58] Sugumaran M., Dali H., Semensi V. (1991). The mechanism of tyrosinase catalyzed oxidative decarboxylation of α-(3,4-dihydroxyphenyl) lactic acid. Biochem. J..

[59] Sugumaran M., Semensi V., Saul S.J. (1989). On the mechanism of oxidation of 3,4-dihydroxyphenethyl alcohol and 3,4-dihydroxyphenyl glycol by cuticular phenoloxidase from *Sarcophaga bullata*. Arch. Insect Biochem. Physiol..

[60] Sugumaran M., Semensi V., Dali H., Saul S.J. (1989). Nonenzymatic transformations of enzymatically generated *N*-acetyldopamine quinone and isomeric dihydrocaffeiyl methylamide quinone. FEBS Lett..

[61] Taylor S.W., Molinski T.F., Rzepecki L.M., Waite H.J. (1991). Oxidation of peptidyl 3,4-dihydroxy phenylalanine analogues: Implications for the biosynthesis of tunichromes and related oligopeptides. J. Nat. Prod..

[62] Sugumaran M., Ricketts D. (1995). Model sclerotization studies. 3. Cuticular enzyme catalyzed oxidation of peptidyl model tyrosine and dopa derivatives. Arch. Insect Biochem. Physiol..

[63] Iverson S.L., Hu L.Q., Vukomanoci V., Bolton J.L. (1995). The influence of the *p*-alkyl substituent on the isomerization of *o*-quinones to *p*-quinone methides: Potential bioactivation mechanism for catechols. Chem. Res. Toxicol..

[64] Bolton J.L., Wu H.W., Hu L.Q. (1996). Mechanism of isomerization of 4-propyl-*o*-quinone to its tautomeric *p*-quinone methide. Chem. Res. Toxicol..

[65] Musson D.G., Karashima D., Rubiero H., Melmon K.L., Cheng A., Castagnoli N. (1980). Synthetic and preliminary hemodynamic and whole animal toxicity studies on (*R*,*S*)-, (*R*)-, and (*S*)-2-methyl-3-(2,4,5-trihydroxyphenyl)alanine. J. Med. Chem..

[66] Sugumaran M., Dali H., Semensi V. (1990). Formation of a stable quinone methide during tyrosinase catalyzed oxidation of α-methyldopa methyl ester and its implication in melanin biosynthesis. Bioorg. Chem..

[67] Crescenzi O., Costantini C., Prota G. (1990). Evidence for the intermediacy of quinone-methides in the rearrangement of aminochromes to 5,6-dihydroxyindoles. Tetrahedron Lett..

[68] Costantini C., Crescenzi O., Prota G. (1991). Mechanism of the rearrangement of dopachrome to 5,6-dihydroxyindole. Tetrahedron Lett..

[69] Palumbo A., d’Ischia M., Misuraca G., Prota G. (1987). Effect of metal ions on the rearrangement of dopachrome. Biochim. Biophys. Acta.

[70] Wakamatsu K., Ito S. (1988). Preparation of eumelanin related metabolites, 5,6-dihydroxyindole, 5,6-dihydroxyindole-2-carboxylic acid, and their *O*-methyl derivatives. Anal. Biochem..

[71] Palumbo A., d’Ischia M., Misuraca G., Prota G. (1989). A new look at the rearrangement of adrenochrome under biomimetic conditions. Biochim. Biophys. Acta.

[72] Manini P., Panzella L., Napolitano A., d’Ischia M. (2007). Oxidation chemistry of norepinephrine: Partitioning of the *o*-quinone between competing cyclization and chain breakdown pathways and their roles in melanin formation. Chem. Res. Toxicol..

[73] Kroesche C., Peter M.G. (1996). Detection of melanochromes by MALDI-TOF mass spectrometry. Tetrahedron.

[74] Al-Kazwini A.T., O’Neill P., Cundall R.B., Admas G.E., Junino A., Maignan J. (1992). Direct observation of the reaction of the quinone methide from 5,6-dihydroxyindole with the nucleophilic azide ion. Tetrahedron Lett..

[75] Al-Kazwini A.T., O’Neill P., Admas G.E., Cundall R.B., Lang G., Junino A. (1991). Reactions of indolic radicals produced upon one-electron oxidation of 5,6-dihydroxyindole and its *N*(1)-methylated analogue. J. Chem. Soc. Perkin Trans. II.

[76] Napolitano A., Corradini M.G., Prota G. (1985). A reinvestigation of the structure of melanochrome. Tetrahedron Lett..

[77] d’Ischia M., Napolitano A., Tsiakas K., Prota G. (1990). New intermediates in the oxidative polymerization of 5,6-dihydroxyindole to melanin promoted by the peroxidase/H_2_O_2_ system. Tetrahedron.

[78] Pezzella A., Panzella L., Crescenzi O., Napolitano A., Navaratman S., Edge R., Land E.J., Barone V., d’Ischia M. (2006). Short-lived quinonoid species from 5,6-dihydroxyindole dimers en route to eumelanin polymers: Integrated chemical, pulse radiolytic, and quantum mechanical investigation. J. Am. Chem. Soc..

[79] Panzella L., Pezzella A., Napolitano A., d’Ischia M. (2007). The first 5,6-dihydroxyindole tetramer by oxidation of 5,5′,6,6′-tetrahydroxy-2,4′-biindolyl and an unexpected issue of positional reactivity en route to eumelanin-related polymers. Tetrahedron Lett..

[80] Pezzella A., Panzella L., Natangelo A., Arzillo M., Napolitano A., d’Ischia M. (2007). 5,6-Dihydroxyindole tetramers with “anomalous” interunit bonding patterns by oxidative coupling of 5,5′,6,6′-Tetrahydroxy-2,7′-biindolyl: Emerging complexities on the way toward an improved model of eumelanin buildup. J. Org. Chem..

[81] Okunda H., Wakamatsu K., Ito S., Sota T. (2008). Possible oxidative polymerization mechanism of 5,6-dihydroxyindole from ab Initio calculations. J. Phys. Chem. A.

[82] Pezzella A., Napolitano A., d’Ischia M., Prota G. (1996). Oxidative polymerization of 5,6-dihydroxy indole-2-carboxylic acid: A new insight. Tetrahedron.

[83] Pezzella A., Vogna D., Prota G. (2002). Atropoisomeric melanin intermediates by oxidation of the melanogenic precursor 5,6-dihydroxyindole-2-carboxylic acid under biomimetic conditions. Tetrahedron.

[84] D’ischia M., Napolitano A., Pezzella A. (2011). 5,6-Dihydroxyindole chemistry: Unexplored opportunities beyond eumelanin. Eur. J. Org. Chem..

[85] Saul S.J., Sugumaran M. (1989). Characterization of a new enzyme system that desaturates the side chain of *N*-acetyldopamine. FEBS Lett..

[86] Saul S.J., Sugumaran M. (1989). *N*-Acetyldopamine quinone methide/1,2-dehydro-*N*-acetyldopamine tautomerase—A new enzyme involved in sclerotization of insect cuticle. FEBS Lett..

[87] Ricketts D., Sugumaran M. (1994). 1,2-dehydro-*N*-β-alanyldopamine as a new intermediate in insect cuticular sclerotization. J. Biol. Chem..

[88] Sugumaran M., Dali H., Semensi V., Hennigan B. (1987). Tyrosinase catalyzed unusual oxidative dimerization of 1,2-dehydro-*N*-acetyldopamine. J. Biol. Chem..

[89] Sugumaran M., Semensi V., Kalyanaraman B., Bruce J.M., Land E.J. (1992). Evidence for the formation of a quinone methide during the oxidation of the insect cuticular sclerotizing precursor, 1.2-dehydro-*N*-acetyldopamine. J. Biol. Chem..

[90] Sugumaran M. (2000). Oxidation chemistry of 1,2-dehydro-N-acetyldopamines: Direct evidence for the formation of 1,2-dehydro-*N*-acetyldopamine quinone. Arch. Biochem. Biophys..

[91] Abebe A., Zheng D., Evans J., Sugumaran M. (2010). Reexamination of the mechanisms of oxidative transformation of the insect cuticular sclerotizing precursor, 1,2-dehydro-*N*-acetyldopamine. Insect Biochem. Mol. Biol..

[92] Sugumaran M., Robinson W. (2012). Structure, biosynthesis and possible function of tunichromes and related compounds. Comp. Biochem. Physiol. B.

[93] Sugumaran M., Robinson W.E. (2010). Bioactive dehydrotyrosyl and dehydrodopyl compounds of marine origin. Mar. Drugs.

[94] Abebe A., Zheng D., Evans J., Sugumaran M. (2016). Novel post-translational oligomerization of peptidyl dehydrodopa model compound, 1,2-dehydro-*N*-acetyldopa methyl ester. Bioorg. Chem..

[95] Napolitano A., Crescenzi C., Prota G. (1993). Copolymerization of 5,6-dihydroxyindole and 5,6-dihydroxy indole-2-carboxylic acid in melanogenesis. Isolation of a cross coupling product. Tetrahedron Lett..

[96] Napolitano A., Pezzella A., Prota G., Seraglia R., Traldi P. (1998). A reassessment of the structure of 5,6-dihydroxyindole-2-carboxylic acid melanins by matrix-assisted laser desorption/ionization mass spectrometry. Rapid Commun. Mass Spectrom..

[97] Bertazzo A., Costa C.V.L., Allegri G., Schiavolin M., Favretto D., Traldi P. (1999). Enzymatic oligomerization of tyrosine by tyrosinase and peroxidase studied by matrix assisted laser desorption/ionization mass spectrometry. Rapid Commun. Mass Spectrom..

[98] Cooksey C.J., Garratt P.J., Land E.J., Pavel S., Ramsden C.A., Riley P.A., Smit N.P.M. (1997). Evidence of the indirect formation of the catecholic intermediate substrate responsible for the autoactivation kinetics of tyrosinase. J. Biol. Chem..

